# Correction to “Long‐term prognosis of patients treated by coronary sinus‐based percutaneous annuloplasty: single centre experience”

**DOI:** 10.1002/ehf2.14540

**Published:** 2023-10-12

Lipiecki, J. , Farhat, H. , Monzy, S. , Caillot, N. , Siminiak, T. , Johnson, T. , Vogt, S. , Stark, M. A. , and Goldberg, S. L. (2020) Long‐term prognosis of patients treated by coronary sinus‐based percutaneous annuloplasty: single centre experience. ESC Heart Failure, 7: 3329–3335. doi:10.1002/ehf2.12955 33047896PMC7755003

In the article by Lipiecki et al. (2020), the name of the second author was published as ‘Hicham Fahrat’.

The correct name of the author should be

Hicham Farhat

We apologize for this error.

